# Impacts of Cytochrome P450 2D6 (CYP2D6) Genetic Polymorphism in Tamoxifen Therapy for Breast Cancer

**DOI:** 10.1055/s-0038-1676303

**Published:** 2018-12

**Authors:** Lucas Soares Bezerra, Marcelo Antônio Oliveira Santos-Veloso, Natanael da Silva Bezerra Junior, Lucilia Carvalho da Fonseca, Wivianne Lisley Andrade Sales

**Affiliations:** 1 Epidemiology and Cardiology Research Group, Department of Health Sciences, Universidade Federal de Pernambuco, Recife, PE, Brazil; 2 School of Medicine, Department of Health Sciences, Faculdade Mauricio de Nassau, Cabo de Santo Agostinho, Recife, PE, Brazil; 3 Department of Gynecology and Obstetrics, Universidade de São Paulo, São Paulo, SP, Brazil

**Keywords:** cytochrome P-450 CYP2D6, polymorphism genetic, tamoxifen, breast neoplasms, therapeutic uses, citocromo P-450 CYP2D6, polimorfismo genético, tamoxifeno, neoplasias da mama, usos terapêuticos

## Abstract

Tamoxifen (TMX) is the main drug used both in pre and postmenopausal women as adjuvant treatment for hormone receptor-positive breast cancer. An important barrier to the use of TMX is the development of drug resistance caused by molecular processes related to genetic and epigenetic mechanisms, such as the actions of cytochrome P450 2D6 (*CYP2D6*) polymorphisms and of its metabolites. The present study aimed to review recent findings related to the impact of *CYP2D6* polymorphisms and how they can affect the results of TMX in breast cancer treatment. The keywords *CYP2D6*, *tamoxifen*, and *breast cancer* were searched in the PubMed, Scopus, The Cochrane Library, Scielo, and Bireme databases. Studies related to other types of neoplasms or based on other isoenzymes from cytochrome P450, but not on CYP2D6, were excluded. The impact of *CYP2D6* polymorphisms in the TMX resistance mechanism remains unclear. The *CYP2D6* gene seems to contribute to decreasing the efficacy of TMX, while the main mechanism responsible for therapy failure, morbidity, and mortality is the progression of the disease.

## Resumo

O tamoxifeno é a principal droga que pode ser utilizada como tratamento hormonal adjuvante em pacientes portadoras de câncer de mama receptor hormonal positivo tanto na pré- quanto na pós-menopausa. Uma das maiores barreiras em seu uso é o desenvolvimento de resistência medicamentosa causada por meio de processos moleculares relacionados a mecanismos genéticos e epigenéticos, como a ação dos polimorfismos do gene citocromo P450 2D6 (*CYP2D6*) e seus metabólitos. O presente estudo busca revisar as descobertas recentes acerca dos impactos dos polimorfismos do gene *CYP2D6* e de como eles podem afetar os resultados do tamoxifeno na terapêutica do câncer de mama. As palavras-chave *CYP2D6*, *tamoxifeno* e *câncer de mama* foram buscadas nas bases de dados Pubmed, Scopus, The Cochrane Library, Scielo e Bireme. Estudos relacionados com outros tipos de câncer ou relacionados a outras isoenzimas do citocromo P450 que não o CYP2D6 foram excluídos. O impacto do polimorfismo do *CYP2D6* nos mecanismos de resistência ao tamoxifeno permanecem controversos. O gene *CYP2D6* parece reduzir a eficácia do TMX; entretanto, os principais fatores associados a falha terapêutica são morbimortalidade e a progressão da doença

## Introduction

Breast cancer is the most common neoplasm among women worldwide, except for skin cancers, accounting for 31% of the cancer cases, and is the second leading cause of cancer-related deaths in females.^1^ ^2^ Breast cancer is an important cause of mortality and morbidity, particularly due to its propensity to metastasize to distant sites such as the liver, the lungs, the brain, and the bones.^3^ ^4^ Many therapies have been studied in the last few years to improve the prognosis and to decrease mortality. In general, adjuvant therapy for breast cancer is used after considering the patient age, tumor staging, biological factors, and tumor volume. These therapies include surgery, hormonal therapy (such as tamoxifen [TMX], toremifene, and raloxifene), anti-HER2 drugs, and chemotherapy.^5^ ^6^

For hormone receptor-positive breast neoplasms, hormonal therapy is mandatory.^6^ ^7^ ^8^ Among the available options, TMX is the main adjuvant hormonal therapy used in pre and postmenopausal patients. Third-generation aromatase inhibitors (anastrozole, letrozole, and exemestane) are only used in postmenopausal patients, but can be used to replace TMX due to their better tolerability and safety.^1^ ^6^ Considering that 70% of the breast neoplasms are estrogen receptor-positive (ER + ), the use of TMX is commonly recommended as the first choice hormonal therapy for breast cancer.^2^ ^5^ ^7^

An important limitation to the use of TMX therapy is the development of drug resistance, which occurs in between 30 and 50% of the cases and may result from complex epigenetic mechanisms, response to stress, inhibition of Bcl-2 family-regulated apoptosis, autophagy, and pharmacologic mechanisms.^2^ ^9^ ^10^ ^11^ The biotransformation of TMX is mediated by isoenzymes from the cytochrome P450, particularly CYP3A4, CYP2B6, CYP2C9, CYP2C19, and CYP2D6; however, the resistance mechanism is also related to phase II metabolism and to ATP-binding cassette (ABC) transporters.^12^ ^13^

The cytochrome P450 family 2 subfamily D member 6 (*CYP2D6*) gene is present in the chromosome 22q13.1 and is responsible for the metabolism of many drugs, such as antidepressants, antipsychotics, and opioids.^5^ ^14^ This enzyme plays a major role in converting TMX into active metabolites, such as 4-hydroxy-N-desmethyltamoxifen (endoxifen), which appears to be inhibited when CYP2D6 inhibitors or TMX are used.^5^ ^12^ ^13^

To date, over 300 variants of the *CYP2D6* gene have been described. According to their different enzyme activities, the *CYP2D6* alleles are classified into functional alleles, reduced function alleles, and non-functional alleles.^12^ ^15^ The type of *CYP2D6* polymorphism varies according to the population analyzed. The gene CYP2D6*4 is more common in Caucasians, CYP2D6*10 in Asian subjects, and CYP2D6*5 in African-Americans.^5^ ^16^ The development of new drugs and therapy adjustments strongly depends on pharmacogenomics studies, which have examined the role of TMX in the physiopathology and in the resistance mechanisms of breast cancer.^17^

The highly polymorphic *CYP2D6* gene encodes the principal enzyme of TMX biotransformation to its major active metabolite, endoxifen.^18^ Some genotypes, such as CYP2D6*4/*4, have been associated with a higher risk of disease relapse and with a lower incidence of adverse drug reactions due to the lower metabolic activation of TMX to endoxifen.^19^ Endoxifen is therefore considered a key metabolite in the modulation of the estrogen receptor pathway.

The association of *CYP2D6* gene polymorphisms with the efficacy of TMX in early-stage breast cancer patients has been assessed in numerous studies, with conflicting results; a notable exception is a complete gene deletion, or alleles with a complete loss of function (such as CYP2D6*4). Some studies have shown no alterations in the efficacy of TMX in patients with breast cancer carrying the *CYP2D6* gene polymorphism in terms of recurrence and overall survival,^12^ ^20^ ^21^ while other studies demonstrated that *CYP2D6* gene polymorphisms (especially *3, *4, and *6) significantly affected the efficacy of TMX.^22^ ^23^ ^24^ Because of these controversial results, available recommendations do not suggest the use of *CYP2D6* genetic testing to select the best endocrine therapy regimen for patients.^18^

Considering previous studies of the role of *CYP2D6* polymorphisms in the TMX resistance mechanism and in the pharmacokinetics of its active metabolite, including *CYP2D6* allele heterogeneity among different populations, the present study was conducted to analyze how mutations in the *CYP2D6* gene affect patients undergoing breast cancer therapy with TMX.^13^ ^25^

## Methods

A narrative review of the presence of *CYP2D6* polymorphisms and how they affect the results of TMX therapy for breast cancer was conducted.

The research was conducted accessing the following databases: PubMed, Scopus, The Cochrane Library, Scielo, and Bireme, using a combination of the terms *CYP2D6*, *tamoxifen*, and *breast cancer*. Only original articles, systematic reviews, and metanalyses published between 1998 and 2018 were included. Articles in English, Spanish, or Portuguese were analyzed.

Studies evaluating the effect of *CYP2D6* polymorphisms in patients with breast neoplasms in all age groups were included. Studies related to other types of neoplasms or based on isoenzymes from the cytochrome P450 other than CYP2D6 were excluded.

The abstract found in the databases were screened by two distinct researchers against the inclusion and exclusion criteria. Full-text articles were retrieved and analyzed to produce the present review. Conflicts were solved by a third independent author.

The results of the present review were grouped according to four main topics: pharmacological properties of TMX, ethnic characteristics of *CYP2D6* alleles, *CYP2D6* polymorphisms and TMX efficacy, and TMX association with other therapies.

### Pharmacological Properties of Tamoxifen

Tamoxifen is considered a selective estrogen receptor modulator (SERM) drug and is a derivative of triphenylethylene.^2^ ^20^ Competition by TMX and its metabolites with estradiol for the occupancy of the estrogen receptor, which blocks estradiol-mediated cellular proliferation, is considered as one of the major mechanisms of the pharmacological action of TMX.^23^ The actions of TMX can be explained by oxidative stress mechanisms that inhibit protein kinase C (PKC), modulate transforming growth factor-β expression, and induce *c-myc* expression, which causes inhibition of malignant cell proliferation by arresting the cell cycle and inducing the apoptosis of breast cancer cells.^10^ ^11^

Similarly to other SERMs, TMX reduces estrogen levels or blocks the estrogen receptor alpha (ERα) signaling pathway.^1^ ^2^ It is well-known that TMX can be used as therapy for many health conditions beyond breast cancer, such as gynecomastia, infertility, osteoporosis, neurodegenerative diseases, retroperitoneal fibrosis, and idiopathic sclerosing mesenteritis.^1^ ^11^ In the endometrial tissue, TMX increases the risk of endometrial cancer by 2.5-fold due to its estrogen agonism and increases the risk of endometrial hyperplasia and of polyp formation.^26^

**Fig. 1:**
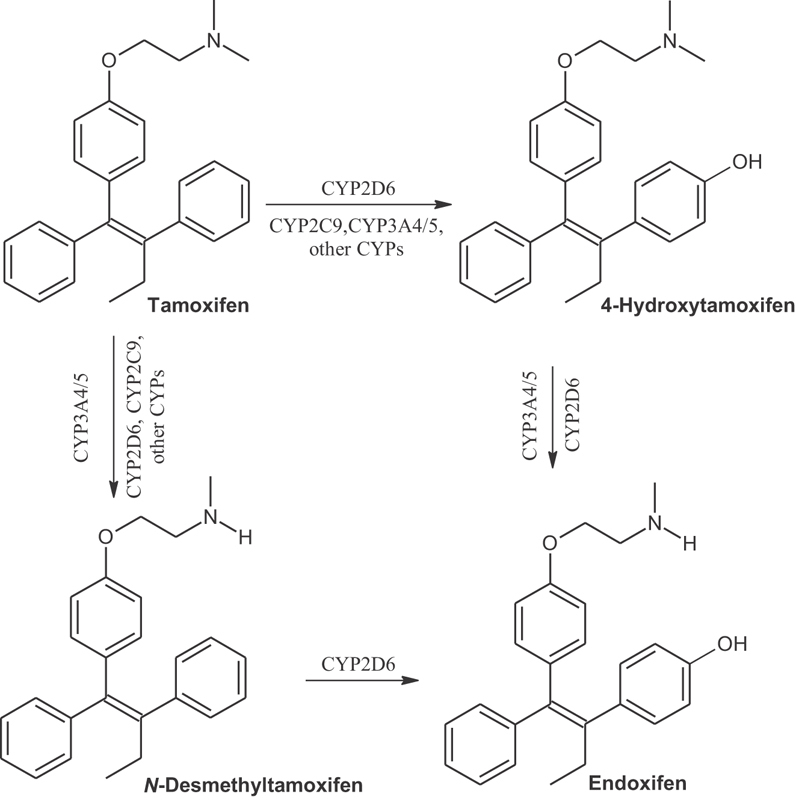
Main tamoxifen active metabolites according to the isoenzymes responsible for its biotransformation process.

Through the action of different enzymes, such as CYP2D6, TMX is converted into three active metabolites: N-Desmethyltamoxifen, 4-Hydroxytamoxifen, and endoxifen (Fig. 1).^1^ ^27^ 4-Hydroxytamoxifen and, particularly, endoxifen, are more potent than TMX itself. Endoxifen is the major molecule responsible for the pharmacological effects of the drug.^5^ ^12^ ^21^ ^27^ The presence of functional *CYP2D6* alleles is required to ensure the presence of high plasma levels of endoxifen.^26^ As demonstrated, endoxifen concentrations are considerably lower in women with lower *CYP2D6* activity.^19^ ^27^

Tamoxifen is metabolized in the liver by specific phase I and phase II enzymes.^26^ ^28^ N-Desmethyltamoxifen is responsible for ∼ 92% of TMX oxidation during the primary metabolism. In the secondary metabolism, the biotransformation of TMX to endoxifen is exclusively catalyzed by CYP2D6 and mainly by the CYP3A subfamily in all other biochemical routes (as shown in Fig. 1).^19^

### Ethnic Characteristics of *CYP2D6* Alleles

According to the literature, the most prevalent variant alleles of the *CYP2D6* gene are comparable between different ethnicities, considering that the sample of patients did not present large variations in their characteristics for each population evaluated (Table 1).^5^ ^14^ ^15^ ^19^ ^21^ ^28^ ^29^ Many ethnic groups remain misrepresented.

**Table 1:** *CYP2D6* most frequent alleles according to different populations

Population	Most recurrent alleles	Study
African and African-Americans	CYP2D6*5/*17	Chin et al. (2016)^15^
Alaska Native	CYP2D6*1	Khan et al. (2018)^21^
American Indian	CYP2D6*1	Khan et al. (2018)^21^
Caucasian	CYP2D6*4	Moyer et al. (2011)^27^
Chinese	CYP2D6*10	Chin et al. (2016)^15^ and Lan et al. (2018)^16^
Malaysian Malay	CYP2D6*10	Chin et al. (2016)^15^
Malaysian Indian	CYP2D6*4/*10	Chin et al. (2016)^15^

In a study evaluating *CYP2D6* polymorphisms in a Chinese population, 778 patients receiving TMX (20 mg/day) or aromatase inhibitors (40 mg/day) were evaluated.^16^ According to the literature, CYP2D6*10 was the most common polymorphism detected, and it was significantly associated with 5-year disease-free survival, which was the time between surgery and recurrence or death in the group who received TMX (325 patients). The age at diagnosis, the T stage, the N stage, the clinical stage, and the tumor grade showed no significant associations.^5^ ^12^ ^16^

According to another study examining an Asian population composed of Chinese, Malay, and Indian subjects (76 of each), the allelic frequencies of CYP2D6*10 and of CYP2D6*5 were respectively 5-fold and 2-fold higher in the healthy Chinese and Malay populations compared with in the Indian population.^29^ In contrast, the Indian and Malay populations showed significantly higher frequencies of CYP2D6*41 compared with the Chinese population.^15^

The *CYP2D6* polymorphisms were evaluated in an American Indian and Alaska Native population in a convenience sample of 380 subjects from the Southcentral Foundation and other 187 subjects from the University of Montana and of the Confederated Salish and Kootenai Tribes.^21^ This cohort study showed that CYP2D6*1 (45.21%) and CYP2D6*2 (26.6%) were the most common alleles identified in the Southcentral Foundation population.

The CYP2D6*41 allele was the most common in the Confederated Salish and Kootenai Tribes population, but showed reduced activity.^21^ The allele induced a non-significant increased plasmatic concentration of N-Desmethyltamoxifen and reduced the plasmatic concentration of endoxifen.^29^

### *CYP2D6* Polymorphisms and Tamoxifen Efficacy

The prevalence of breast cancer was found to be associated with *CYP2* gene polymorphisms in a study examining the prevalence of the most common allelic variants from *CYP2D6* (CYP2D6*4, CYP2D6*10, CYP3A5*3, and CYP2C19*2).^12^ In 128 patients with a diagnosis of breast cancer, a significant difference in the frequencies of genotype was found between the therapy and control groups (*p* = 0.01); the prevalent genotypes were AA (88%) on CYP3A5*3, GG (88%) on CYP2C19*2, CC (75%) on CYP2D6*10, and EM (79%) on CYP2D6*4.

The *CYP2D6* gene was the major contributor to significant associations between genetic variation and TMX metabolism.^21^ ^27^ ^29^ The *CYP2D6* variation was significantly associated with plasma endoxifen (*p* = 0.0010) levels and with the endoxifen/TMX metabolic ratio (*p* = 4.4 × 10^−7^), particularly CYP2D6*5 and CYP2D6*10.^21^ ^29^ Caucasian patients carrying the CYP2D6*4 allele had a significantly increased risk of recurrence of breast cancer.^25^ Two large studies found no association between the *CYP2D6* genotype and clinical outcome, revealing controversial results for the role of *CYP2D6* in the TMX resistance mechanism.^16^ ^28^

A few studies found no significant difference between poor, intermediate, and extensive metabolizer genotypes and recurrence or metastasis. However, CYP2D6*10/*10 and heterozygous null alleles appeared to decrease the metabolism of TMX and to produce less pharmacologically active TMX metabolites, resulting in a higher risk of recurrence and metastasis.^25^ ^30^

Zembutsu et al^31^ reported a significant effect of the *CYP2D6* polymorphism on the response to TMX therapy in the first prospective study on this topic by examining a sample of 279 individuals. The clinical response in breast cancer tissues, measured as the K_i_-67 expression levels, was significantly associated with TMX therapy. Particularly, patients with homozygous variant alleles showed a lower K_i_-67 response than those carrying at least one wild-type allele.^31^

Divergent results were also described. A recent retrospective cohort of 957 subjects found no evidence of decreased TMX effectiveness in patients with *CYP2D6* polymorphism phenotypes.^24^ In contrast, after adjusting for clinical variables, a positive association between low *CYP2D6* activity and superior TXM treatment outcomes was found, which is consistent with the results of two previous studies.^22^ ^23^

An international randomized, phase III double-blind trial obtained tumor tissues and isolated the DNA from 4,861 postmenopausal women with hormone receptor–positive breast cancer and found no association between *CYP2D6* metabolism phenotypes and breast cancer-free interval among patients receiving tamoxifen monotherapy; effects were only observed in those who had undergone previous chemotherapy (*p* = 0.35).^32^

In a study of a Brazilian population, an analysis of a cohort of 92 patients with hormone-sensitive breast carcinoma showed no significant association between phenotypes of intermediate and null metabolizers with breast cancer recurrence.^33^ The authors hypothesized that the lack of significance was related to the small patient sample, which is the main critique of previous studies showing a negative association. Table 2 summarizes the association measurements for the respective studies included in the present review.

**Table 2:** Association measurements for studies analyzing the effects of *CYP2D6* on tamoxifen outcomes

Author, Year	Country	Ethnicity	Major Variant Allele	RR/OR/HR	95% CI	Sample size
Hertz et al. (2017)^24^	USA	Caucasian	*2; *4; *41	RR 0.44	0.22–0.98	957
Teh et al. (2011)^25^	Malaysia	Malaysian Malay	*10	OR 9.9	2.11–46.6	95
Lan et al. (2018)^16^	China	Chinese	*10	HR 1.87	1.19–2.93	778
Wegman et al. (2005)^22^	Sweden	Caucasian	*4	RR 0.28	0.21–1.12	226
Nowell et al. (2005)^23^	USA	Caucasian, African-Americans	*4	HR 0.77	0.32–1.81	337
Motamedi et al. (2013)^30^	Iran	*-*	*-*	OR 1.6‡	0.53–4.78	79

Abbreviations: CI, confidence interval; HR, hazard ratio; OR, odds ratio; RR, relative risk; USA, United States of America.

‡ OR for tamoxifen resistance for the presence of three *CYP2D6* copies.

### Tamoxifen Association with Other Therapies

Most studies utilized only a dose of 20 mg/day of TMX to evaluate its effects on breast cancer. However, according to Facina et al (2003),^34^ a dose of 10 mg/day had antiproliferative effects on the mammary epithelium adjacent to the fibroadenoma in premenopausal women. Other studies did not describe the dose of TMX administered.^13^

In a phase II clinical trial known as TAMRAD (tamoxifen plus everolimus),^9^ a comparison between everolimus (10 mg/day) plus TMX (20 mg/day) and TMX (20 mg/day) was performed. The study suggested an improvement in the overall survival following the combination treatment compared with treatment with TMX alone (*p* = 0.007).^9^ ^35^ However, only 20% of the patients required a reduction on the dose of everolimus due to adverse effects, such as stomatitis, rash, thrombocytopenia, and pneumonitis. The study showed that TMX alone was not strongly associated with the development of adverse effects.^9^

Tamoxifen (20 mg/day) therapy was associated with significantly increased overall survival compared with fulvestrant (250 mg/day) as the first-line therapy (hazard ratio = 1.29; 95% CI: 1.01–1.64; *p* = 0.04).^35^

Three other studies evaluated the administration of TMX (20 mg/day) versus anastrozole (1 mg/day) and found no difference in the mortality range.^36^ ^37^ ^38^ Additionally, both drugs showed equivalent efficacies as first-line therapies for advanced breast cancer in postmenopausal women. Flushes, nausea, asthenia, and pain were the most common adverse effects and treatment failure was mainly explained by the progression of the disease.^36^ ^37^

## Limitations of the Studies

Although studies have demonstrated the favorable role of the *CYP2D6* gene in TMX resistance, there were some limitations such as the lack of a description of the TMX dose and of information regarding the adherence to the therapy, inadequate duration, and omissions of concomitant drugs used to treat breast cancer. Additionally, other associated pathologies that may have decreased the effects of TMX were not reported. Moreover, most clinical studies evaluated Asian populations only; therefore, the results may not be applicable to other populations.

Additional studies of the tumor and genome-related resistance mechanism to treatments will facilitate the development of better therapies and will increase the disease-free survival rate and the quality of life of the patients. There is insufficient information to consider the *CYP2D6* genotype as a biomarker for predicting TMX efficacy. Larger clinical studies are necessary, particularly considering the ethnic particularities of the *CYP2D6* polymorphisms.

## Conclusion

Hormonal treatment with TMX had advantages compared with other hormonal drugs on hormone receptor-positive breast cancer, particularly considering that it is the main option for both pre- and postmenopausal women. The effects of *CYP2D6* polymorphisms on the TMX resistance mechanism remain unclear. The *CYP2D6* gene appears to contribute to decreasing the efficiency of TMX, while the main mechanism responsible for therapy failure, morbidity, and mortality is the progression of the disease.

## References

[1] Shagufta A I Ahmad I Tamoxifen a pioneering drug: An update on the therapeutic potential of tamoxifen derivatives Eur J Med Chem 2018 143 515 531 Doi: 10.1016/j.ejmech.2017.11.056 29207335 10.1016/j.ejmech.2017.11.056

[2] Jahangiri R Mosaffa F Emami Razavi A Teimoori-Toolabi L Jamialahmadi K Altered DNA methyltransferases promoter methylation and mRNA expression are associated with tamoxifen response in breast tumors J Cell Physiol 2018 233 09 7305 7319 Doi: 10.1002/jcp.26562 29574992 10.1002/jcp.26562 PMC13482140

[3] Shah N Mohammad A S Saralkar P Investigational chemotherapy and novel pharmacokinetic mechanisms for the treatment of breast cancer brain metastases Pharmacol Res 2018 132 47 68 Doi: 10.1016/j.phrs.2018.03.021 29604436 10.1016/j.phrs.2018.03.021 PMC5997530

[4] Allan A L Vantyghem S A Tuck A B Chambers A F Tumor dormancy and cancer stem cells: implications for the biology and treatment of breast cancer metastasis Breast Dis 2006–2007–2007 26 87 98 Doi: 10.3233/BD-2007-26108 10.3233/bd-2007-26108 17473368

[5] Souza R DM Martins D MF Chein M BC Brito L MO Importância do CYP2D6 em usuárias de tamoxifeno no câncer de mama Femina 2011 39 267 274

[6] Drăgănescu M Carmocan C Hormone therapy in breast cancer Chirurgia (Bucur) 2017 112 04 413 417 Doi: 10.21614/chirurgia.112.4.413 28862117 10.21614/chirurgia.112.4.413

[7] Abdel-Hafiz H A Epigenetic mechanisms of tamoxifen resistance in luminal breast cancer Diseases 2017 5 03 E16 Doi: 10.3390/diseases5030016 10.3390/diseases5030016 PMC5622332 28933369

[8] Fleeman N Payne K Newman W G Are health technology assessments of pharmacogenetic tests feasible? A case study of CYP2D6 testing in the treatment of breast cancer with tamoxifen Per Med 2013 10 06 601 611 Doi: 10.2217/pme.13.60 29776195 10.2217/pme.13.60

[9] Bachelot T Bourgier C Cropet C Randomized phase II trial of everolimus in combination with tamoxifen in patients with hormone receptor-positive, human epidermal growth factor receptor 2-negative metastatic breast cancer with prior exposure to aromatase inhibitors: a GINECO study J Clin Oncol 2012 30 22 2718 2724 Doi: 10.1200/JCO.2011.39.0708 22565002 10.1200/JCO.2011.39.0708

[10] Bekele R T Venkatraman G Liu R Z Oxidative stress contributes to the tamoxifen-induced killing of breast cancer cells: implications for tamoxifen therapy and resistance Sci Rep 2016 6 21164 Doi: 10.1038/srep21164 26883574 10.1038/srep21164 PMC4756695

[11] Viedma-Rodríguez R Baiza-Gutman L Salamanca-Gómez F Mechanisms associated with resistance to tamoxifen in estrogen receptor-positive breast cancer (review) Oncol Rep 2014 32 01 3 15 Doi: 10.3892/or.2014.3190 24841429 10.3892/or.2014.3190

[12] Thota K Prasad K Basaveswara Rao M V Detection of cytochrome p450 polymorphisms in breast cancer patients may impact on tamoxifen therapy Asian Pac J Cancer Prev 2018 19 02 343 350 Doi: 10.22034/APJCP.2018.19.2.343 29479969 10.22034/APJCP.2018.19.2.343 PMC5980918

[13] Hansten P D The Underrated Risks of Tamoxifen Drug Interactions Eur J Drug Metab Pharmacokinet 2018 43 05 495 508 Doi: 10.1007/s13318-018-0475-9 29637493 10.1007/s13318-018-0475-9 PMC6133076

[14] Lyon E Gastier Foster J Palomaki G E Laboratory testing of CYP2D6 alleles in relation to tamoxifen therapy Genet Med 2012 14 12 990 1000 Doi: 10.1038/gim.2012.108 22955113 10.1038/gim.2012.108

[15] Chin F W Chan S C Abdul Rahman S Noor Akmal S Rosli R CYP2D6 Genetic polymorphisms and phenotypes in different ethnicities of Malaysian breast cancer patients Breast J 2016 22 01 54 62 Doi: 10.1111/tbj.12518 26510986 10.1111/tbj.12518

[16] Lan B Ma F Zhai X The relationship between the CYP2D6 polymorphisms and tamoxifen efficacy in adjuvant endocrine therapy of breast cancer patients in Chinese Han population Int J Cancer 2018 143 01 184 189 Doi: 10.1002/ijc.31291 29396856 10.1002/ijc.31291

[17] Wellmann R Borden B A Danahey K Analyzing the clinical actionability of germline pharmacogenomic findings in oncology Cancer 2018 124 14 3052 3065 Doi: 10.1002/cncr.31382 29742281 10.1002/cncr.31382 PMC6354920

[18] Del Re M Rofi E Citi V Fidilio L Danesi R Should CYP2D6 be genotyped when treating with tamoxifen? Pharmacogenomics 2016 17 18 1967 1969 Doi: 10.2217/pgs-2016-0162 27883289 10.2217/pgs-2016-0162

[19] Goetz M P Kamal A Ames M M Tamoxifen pharmacogenomics: the role of CYP2D6 as a predictor of drug response Clin Pharmacol Ther 2008 83 01 160 166 Doi: 10.1038/sj.clpt.6100367 17882159 10.1038/sj.clpt.6100367 PMC2752373

[20] Monteagudo E Gándola Y González L Bregni C Carlucci A M Development, characterization, and in vitro evaluation of tamoxifen microemulsions J Drug Deliv 2012 2012 236713 Doi: 10.1155/2012/236713 22272375 10.1155/2012/236713 PMC3261494

[21] Khan B A Robinson R Fohner A E Cytochrome P450 genetic variation associated with tamoxifen biotransformation in American Indian and Alaska native people Clin Transl Sci 2018 11 03 312 321 Doi: 10.1111/cts.12542 29436156 10.1111/cts.12542 PMC5944577

[22] Wegman P Vainikka L Stål O Genotype of metabolic enzymes and the benefit of tamoxifen in postmenopausal breast cancer patients Breast Cancer Res 2005 7 03 R284 R290 Doi: 10.1186/bcr993 15987423 10.1186/bcr993 PMC1143572

[23] Nowell S A Ahn J Rae J M Association of genetic variation in tamoxifen-metabolizing enzymes with overall survival and recurrence of disease in breast cancer patients Breast Cancer Res Treat 2005 91 03 249 258 Doi: 10.1007/s10549-004-7751-x 15952058 10.1007/s10549-004-7751-x

[24] Hertz D L Kidwell K M Hilsenbeck S G CYP2D6 genotype is not associated with survival in breast cancer patients treated with tamoxifen: results from a population-based study Breast Cancer Res Treat 2017 166 01 277 287 Doi: 10.1007/s10549-017-4400-8 28730340 10.1007/s10549-017-4400-8 PMC6028015

[25] Teh L K Mohamed N I Salleh M Z The risk of recurrence in breast cancer patients treated with tamoxifen: polymorphisms of CYP2D6 and ABCB1 AAPS J 2012 14 01 52 59 Doi: 10.1208/s12248-011-9313-6 22183189 10.1208/s12248-011-9313-6 PMC3291182

[26] Dean L Tamoxifen therapy and CYP2D6 genotype Bethesda, MD National Center for Biotechnology Information 2014 1 12 https://www.ncbi.nlm.nih.gov/books/NBK247013/pdf/Bookshelf_NBK247013.pdf. Accessed November 12, 2017. 28520357

[27] Moyer A M Suman V J Weinshilboum R M SULT1A1, CYP2C19 and disease-free survival in early breast cancer patients receiving tamoxifen Pharmacogenomics 2011 12 11 1535 1543 Doi: 10.2217/pgs.11.97 21961651 10.2217/pgs.11.97 PMC3235041

[28] Kiyotani K Mushiroda T Nakamura Y Zembutsu H Pharmacogenomics of tamoxifen: roles of drug metabolizing enzymes and transporters Drug Metab Pharmacokinet 2012 27 01 122 131 Doi: 10.2133/dmpk.DMPK-11-RV-084 22041137 10.2133/dmpk.dmpk-11-rv-084

[29] Lim J SL Chen X A Singh O Impact of CYP2D6, CYP3A5, CYP2C9 and CYP2C19 polymorphisms on tamoxifen pharmacokinetics in Asian breast cancer patients Br J Clin Pharmacol 2011 71 05 737 750 Doi: 10.1111/j.1365-2125.2011.03905.x 21480951 10.1111/j.1365-2125.2011.03905.x PMC3093079

[30] Motamedi S Majidzadeh K Mazaheri M Anbiaie R Mortazavizadeh S MR Esmaeili R Tamoxifen resistance and CYP2D6 copy numbers in breast cancer patients Asian Pac J Cancer Prev 2012 13 12 6101 6104 23464412 10.7314/apjcp.2012.13.12.6101

[31] Zembutsu H Nakamura S Akashi-Tanaka S Significant effect of polymorphisms in CYP2D6 on response to tamoxifen therapy for breast cancer: a prospective multicenter study Clin Cancer Res 2017 23 08 2019 2026 Doi: 10.1158/1078-0432.CCR-16-1779 27797974 10.1158/1078-0432.CCR-16-1779

[32] Regan M M Leyland-Jones B Bouzyk M CYP2D6 genotype and tamoxifen response in postmenopausal women with endocrine-responsive breast cancer: the breast international group 1-98 trial J Natl Cancer Inst 2012 104 06 441 451 Doi: 10.1093/jnci/djs125 22395644 10.1093/jnci/djs125 PMC3309132

[33] De Ameida Melo M De Vasconcelos-Valença R J Neto F M CYP2D6 gene polymorphisms in Brazilian patients with breast cancer treated with adjuvant tamoxifen and its association with disease recurrence Biomed Rep 2016 5 05 574 578 Doi: 10.3892/br.2016.771 27882219 10.3892/br.2016.771 PMC5103674

[34] Facina G Baracat E C Lima G R Gebrim L H Effects of different tamoxifen doses on mammary epithelium proliferation Rev Bras Ginecol Obstet 2003 25 185 191 Doi: 10.1590/S0100-72032003000300007

[35] Reinert T Barrios C H Overall survival and progression-free survival with endocrine therapy for hormone receptor-positive, HER2-negative advanced breast cancer: reviewreviewTher Adv Med Oncol 2017 9 11 693 709 Doi: 10.1177/1758834017728928 29344106 10.1177/1758834017728928 PMC5764151

[36] Bonneterre J Thürlimann B Robertson J F Anastrozole versus tamoxifen as first-line therapy for advanced breast cancer in 668 postmenopausal women: results of the Tamoxifen or Arimidex Randomized Group Efficacy and Tolerability study J Clin Oncol 2000 18 22 3748 3757 Doi: 10.1200/JCO.2000.18.22.3748 11078487 10.1200/JCO.2000.18.22.3748

[37] Bonneterre J Buzdar A Nabholtz J M Anastrozole is superior to tamoxifen as first-line therapy in hormone receptor positive advanced breast carcinoma Cancer 2001 92 09 2247 2258 Doi: 10.1002/1097-0142(20011101)92:9<2247::AID-CNCR1570>3.0.CO;2-Y 11745278 10.1002/1097-0142(20011101)92:9<2247::aid-cncr1570>3.0.co;2-y

[38] Nabholtz J M Bonneterre J Buzdar A Robertson J F Thürlimann B Anastrozole (Arimidex) versus tamoxifen as first-line therapy for advanced breast cancer in postmenopausal women: survival analysis and updated safety results Eur J Cancer 2003 39 12 1684 1689 Doi: 10.1016/S0959-8049(03)00326-5 12888362 10.1016/s0959-8049(03)00326-5

